# RNA Binding Proteins As Regulators of Oxidative Stress Identified by a Targeted CRISPR-Cas9 Single Guide RNA Library

**DOI:** 10.1089/crispr.2020.0116

**Published:** 2021-06-16

**Authors:** David J. Turner, Martin Turner

**Affiliations:** Laboratory of Lymphocyte Signalling and Development, The Babraham Institute, Babraham Research Campus, Cambridge, United Kingdom.

## Abstract

The clustered regularly interspaced short palindromic repeats (CRISPR)-Cas9 genome editing system has been broadly adopted for high-throughput genetic screens. However, the application of genome-wide single guide RNA (sgRNA) libraries can be challenging. We generated a custom sgRNA library, an order of magnitude smaller than genome-wide alternatives, to facilitate the genetic screening of RNA binding proteins (RBPs). We demonstrated the utility of our reagent in a genetic screen for RBPs that conveyed cellular resistance or sensitivity to oxidative stress induced by paraquat. This identified that CSDE1 and STRAP, proteins that interact with each other, convey sensitivity to oxidative stress and that Pumilio homologues (PUM1 and PUM2) convey resistance. Targeting eIF4-E1 and -A1 protected cells from high-dose paraquat, whereas eIF4E2 targeted cells did less well. We also found that G3BP1 promoted sensitivity to a low dose of paraquat but protected cells at a higher dose. Our study highlights the use of genetic screens to identify roles of RBPs and identifies novel genes regulating sensitivity to oxidative stress.

## Introduction

Functional genomics screens have been facilitated by the greater specificity and on-target efficiency of clustered regularly interspaced short palindromic repeats (CRISPR)-Cas9-mediated gene targeting over alternative approaches such as small interfering RNA or small hairpin RNA-mediated gene knockdown. However, genome-wide genetic screens, with libraries encompassing tens of thousands of guide RNAs (gRNAs), commonly have type II errors that limits the identification of novel genes mediating modest effects in a biological assay. Therefore, targeted libraries that address a particular area of cellular biology can provide a more sensitive screening option. For example, a library targeting ∼3000 metabolic enzymes and their regulators identified CD8 T cells deficient for the RNA binding protein (RBP) Regnase-1 have prolonged survival, and more robust effector function in the tumor microenvironment.^1^

Superoxide and hydrogen peroxide are forms of reactive oxygen species (ROS) that act as essential intracellular secondary messengers in many cell types including innate and adaptive immune cells.^2^ ROS are essential for toll-like receptor signaling pathways and for the bactericidal activity of macrophages.^3^ In lymphocytes, ROS increase upon activation by antigen and further promote activation and proliferation.^4^ ROS potentiate signaling pathways by oxidizing cysteine residues in the active sites of phosphatases leading to their inactivation. Hence, ROS can reduce the activation threshold for signaling pathways that are suppressed by phosphatases, as well as prolong their activation. This is exemplified by the inactivation of the lipid phosphatase known as Phosphatase and tensin homologue (PTEN) by ROS and the consequent activation of the PI(3)K/AKT cascade by ROS.^5^

If the production of ROS exceeds the capacity of antioxidant defenses, this leads to oxidative stress that can cause lipid peroxidation, enzymatic inactivation, DNA damage, and ultimately apoptosis.^6^ Cells have evolved extensive mechanisms to remove or mitigate the effects of oxidative stress. These include cytosolic superoxide dismutase (SOD1) and mitochondrial SOD2 enzymes that rapidly convert superoxide into the weaker oxidizing agent hydrogen peroxide.^6^ Hydrogen peroxide can be reduced to water plus molecular oxygen by catalase, or by reduced glutathione or thiol-containing enzymes such as thioredoxin, peroxiredoxins, and glutaredoxins.^6^ Exposure to the chemical herbicide paraquat can induce oxidative stress, as paraquat catalyzes the partial reduction of molecular oxygen (O_2_) by redox enzymes, leading to the direct intracellular formation of superoxide-free radicals and the indirect generation of hydrogen peroxide.^7,8^ Oxidative stress triggers an arrest of translation initiation and the dissociation of messenger RNA (mRNA) from polyribosomes that reduces the energetic demand of high rates of translation and leads to the selective translation of stress-induced genes.^9^

The global translational response to stress induces the formation in the cytoplasm of microscopically visible structures called stress granules (SGs) comprising RBPs and nontranslating mRNA.^10^ SG formation is promoted by intermolecular RNA–RNA interactions formed by nontranslating mRNA.^11,12^ Such RNA condensation can be inhibited by eIF4A1.^13^ RBPs also nucleate the formation of SGs, for example, the multimerization of the RBP G3BP1 initiates SG formation.^14^ In unstressed cells, G3BP1 binds USP10 and blocks its antioxidant function.^15^ Upon stress, CAPRIN1 binds G3BP1 and replaces USP10, this process promotes SG formation and uncovers the antioxidant activity of USP10.^15,16^

In this study, we generated a targeted single guide RNA (sgRNA) library against 724 human RBPs and used this in a genetic screen to identify RBPs that conveyed cellular resistance or sensitivity to high or low concentrations of paraquat. The screen identified expected targets, including USP10 that was enriched for a role in mediating resistance to paraquat. Both G3BP1 and CAPRIN1 enriched for a role in mediating sensitivity to low concentrations of paraquat but switched to convey resistance at the higher dose. Furthermore, our screen identified novel RBPs that convey sensitivity or resistance to paraquat.

## Results

### Generation of a human RBP sgRNA library

**FIG. 1.**
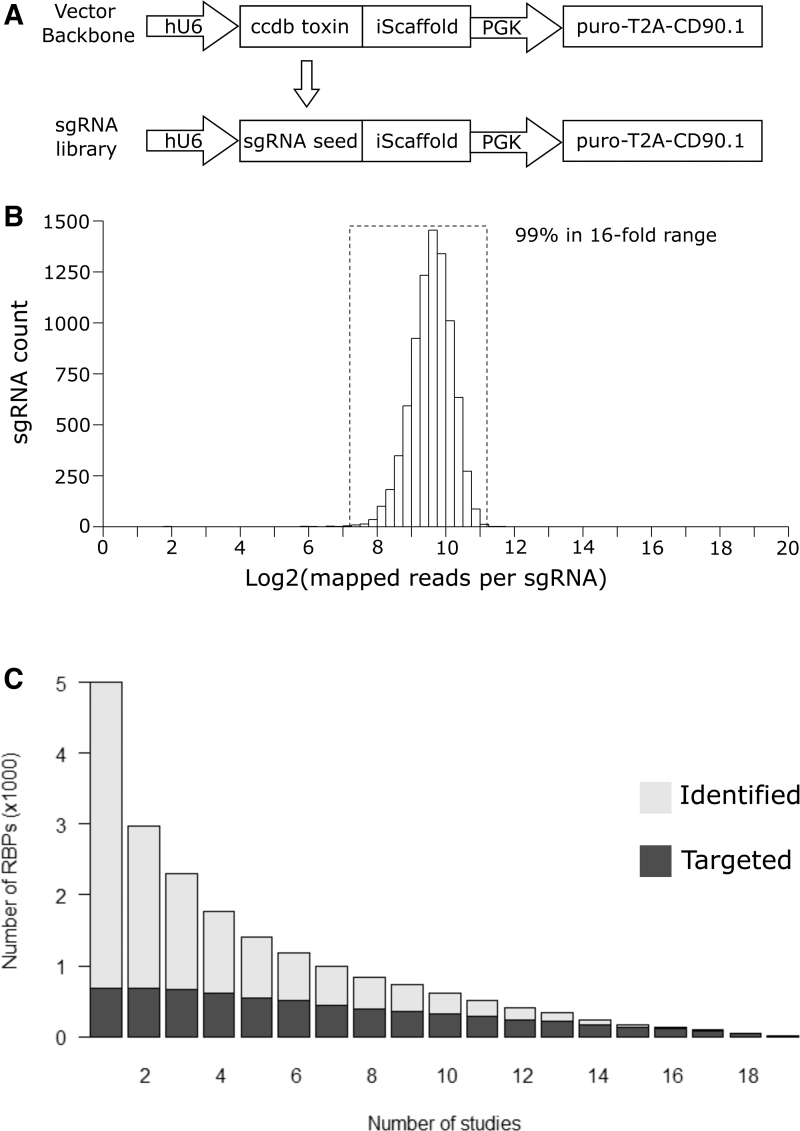
Generation of an sgRNA library for human RBPs. **(A)** Schematic of vector backbone and cloning strategy of the sgRNA library. **(B)** Distribution of the representations of sgRNAs in our library. **(C)** Summary of the number of putative RBPs identified by at least  × number of high-throughput studies in gray. Summary of the number of RBPs targeted by our human sgRNA library and identified by at least × number of high-throughput studies in black. RBPs, RNA binding proteins; sgRNA, single guide RNA.

To facilitate targeted genetic screens, we focused on messenger RNA binding proteins (mRBPs). We compiled a list of 725 putative human mRBPs informed by a rigorous manual curation by Tuschl and colleagues^17^ and mRNA interactome capture studies^18–20^ (Supplementary Table S1). We created a library of lentiviral vectors encoding 8260 sgRNAs targeting these RBPs (Supplementary Table S2). In addition, our library contains 500 negative control sgRNAs with no complementary genomic sequence and targets 51 positive control genes selected for known roles in biological processes. The library contains 10 sgRNAs per gene targeted to maximize the statistical power to distinguish true positives from potential false positives. The lentivirus vector enables selection of transduced cells either by puromycin resistance or by the cell surface antigen CD90.1 (Thy1.1), which allows the identification of transduced cells by flow cytometry and their separation by fluorescence or magnetic based cell sorting. To generate the custom library, a pool of double-stranded DNA (dsDNA) encoding sgRNA seed sequences was introduced into the backbone vector to replace the ccdB toxin gene that was included in the parental vector design to prevent library contamination by plasmids lacking guides (Fig. 1A).

We validated the generation of our sgRNA library by next-generation sequencing (Fig. 1B). This demonstrated that the representation of sgRNAs within the library was normally distributed, that all designed sgRNAs were present, and that the library has a tight distribution with >99.9% of the sgRNAs within a 16-fold range. High-throughput studies employing RNA interactome capture or orthogonal phase separation have annotated RBPs in different contexts (Supplementary Table S3). These implicate ∼5000 proteins as RBPs, of which ∼3000 have been identified at least twice and ∼2000 have been identified by at least three studies (Columns 1–3, Fig. 1C). Our library targets 725 of these RBPs and is skewed toward those identified most frequently by high-throughput approaches (Fig. 1C).

### Validation of cell line and paraquat toxicity assay

**FIG. 2.**
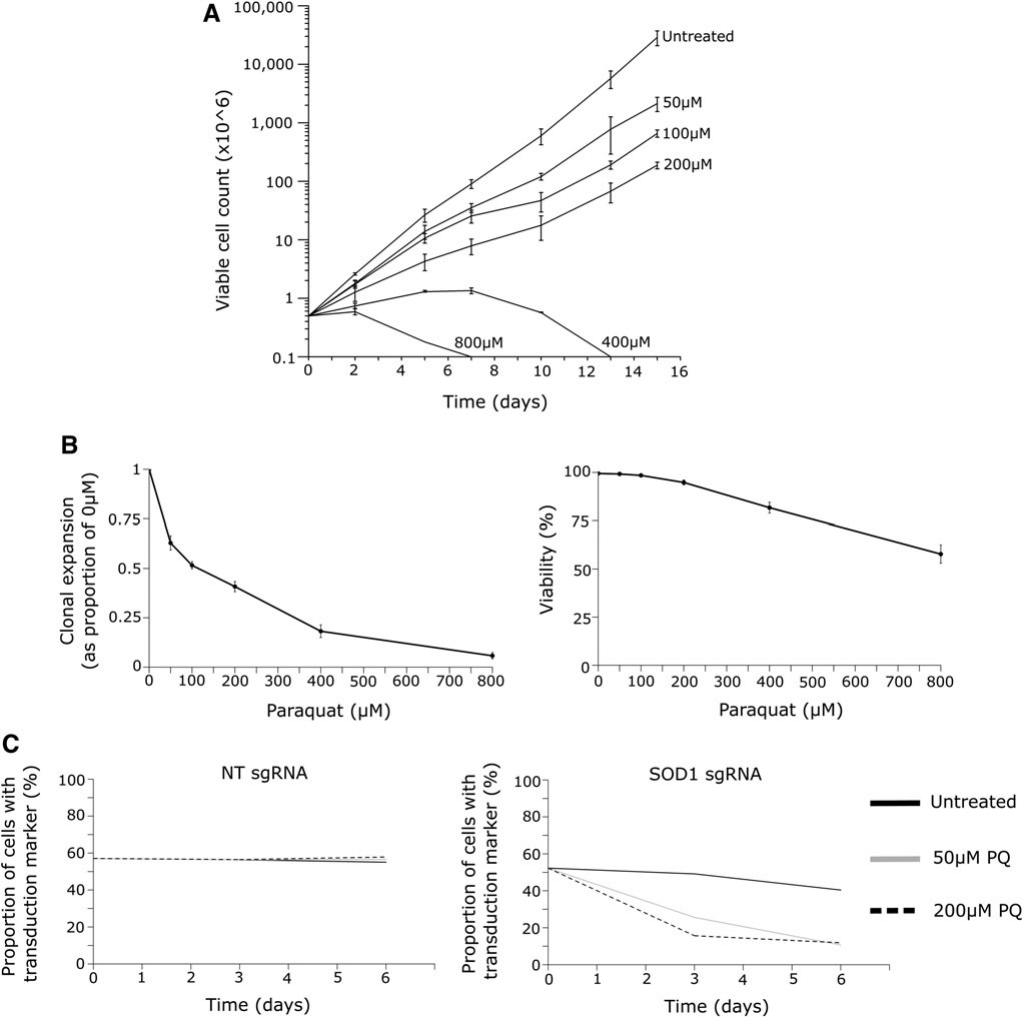
Validation of a PQ toxicity assay. **(A)** Cell count of Jurkat cells exposed to a titration of PQ over a 15-day time course. Error bars represent SEM for three technical replicates. **(B)** Left: Clonal expansion of Jurkat cells in PQ normalized to the untreated condition. Right: Viability of Jurkat cells in PQ. Measurements after 3 days of exposure. Error bars represent SEM for three technical replicates. **(C)** Competition assay of either *SOD1* KO cells or NT control cells cultured with wild-type cells in the presence of PQ. This is representative of three experiments, each performed with three *SOD1* sgRNAs, which all had a consistent phenotype. NT, nontargeting; PQ, paraquat; SEM, standard error of the mean.

We engineered the human Jurkat acute T cell leukemic cell line to express Cas9. We confirmed efficient DNA editing by a clonal line using sgRNAs targeting the ELAVL1 gene (Supplementary Fig. S1). To identify appropriate conditions for a genetic screen, Jurkat cells were cultured for a 2-week time course with titrated amounts of paraquat. We observed that high concentrations (≥400 μM) of paraquat induced cell death (Fig. 2A), whereas at lower concentrations (≤200 μM), there was a dose-dependent reduction in the rate of growth without a large effect on viability (Fig. 2B). This pilot study indicated that between 50 and 200 μM would be an appropriate range of paraquat concentration to identify RBPs affecting sensitivity and resistance of Jurkat cells to oxidative stress.

To validate this further, we performed the CRISPR-Cas9-mediated gene knockout of *SOD1*. *SOD1* KO Jurkat cells were outcompeted by unmodified Jurkat cells in the same culture at both 50 and 200 μM of paraquat over a 6-day time course (Fig. 2C). This was not the case in the absence of paraquat, nor was it the case when Jurkat cells were transduced with a lentivirus producing a nontargeting gRNA (Fig. 2C). These Jurkat Cas9 cells are thus a suitable system to identify regulators of paraquat toxicity.

### Genetic screen of RBPs involved in paraquat toxicity

**FIG. 3.**
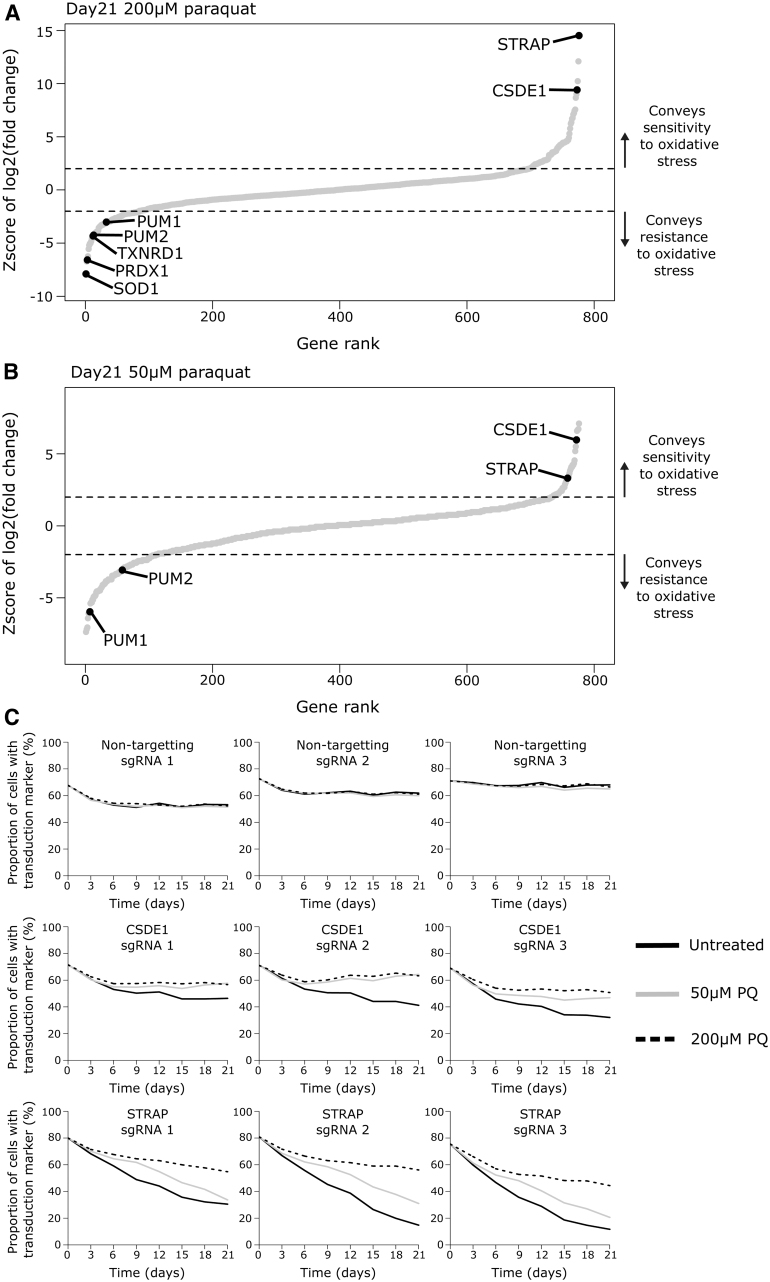
CRISPR-Cas9 gene knockout screen of RBPs involved in oxidative stress resistance or sensitivity. **(A)** Enrichment of RBPs and positive control genes in 200 μM paraquat screen compared with the condition without paraquat treatment at day 21. **(B)** Enrichment of RBPs in 50 μM paraquat screen compared with the condition without paraqaut treatment at day 21. Select RBPs highlighted. Dashed line represents 2 standard deviations of the distribution of nontargeting sgRNAs from the mean representation of nontargeting sgRNAs. This is the cutoff for statistical significance. **(C)** Validation that the genetic knockout of *CSDE1* or *STRAP* in Jurkat cells conveys resistance to paraquat at 50 or 200 μM. CRISPR, custered regularly interspaced short palindromic repeats.

We performed CRISPR-Cas9 gene knockout screens in the absence of paraquat and at concentrations of 50 and 200 μM with readouts after 14 and 21 days. These conditions were chosen as we did not expect the phenotype of the knockout of an RBP to be greater than that of the *SOD1* KO. The data sets generated at day 14 (Supplementary Tables S4 and S5) and day 21 (Supplementary Tables S6 and S7) overlapped with each other (Spearman's rho is 0.679 for 50 μM and 0.841 for 200 μM), with the same RBPs enriched at both days. However, the number and statistical significance of the hits at day 21 are greater, therefore, we concentrate on this data set. The technical success of our genetic screen was exemplified by the positive control genes. Superoxide dismutase (*SOD1*) was the top ranked gene for mediating paraquat resistance at 200 μM paraquat at day 21 (Fig. 3A) and day 14. Furthermore, thioredoxin (*TXNRD1*) and peroxiredoxin (*PRDX1*) were also among the top hits (Fig. 3A). Analysis of the genetic screen in the absence of paraquat identified RBPs that promote the proliferation and/or survival of Jurkat cells (Supplementary Table S8). RBPs such as PABPC1 and the SF3B family of splicing factors were critical for Jurkat proliferation or survival but they did not show enrichment in the presence of oxidative stress (Supplementary Fig. S2). This demonstrates that the genetic screen identifies regulators of oxidative stress independently of their contribution to the proliferation or survival of Jurkat cells in the absence of stress.

In the 50 μM paraquat condition, 108 RBPs enriched for a role in conferring resistance and 41 enriched for a role in conferring sensitivity to oxidative stress at day 21. In the 200 μM paraquat condition, 157 RBPs were enriched with 78 conferring resistance and 79 conferring sensitivity to oxidative stress at day 21. In total, 15 RBPs had divergent enrichment between the genetic screens performed in low and high paraquat conditions (Supplementary Table S9).

The RBPs CSDE1 and STRAP enriched among the top hits for mediating sensitivity to paraquat in both the high (Fig. 3A) and low (Fig. 3B) paraquat concentrations at day 21. These results were validated with three different gRNAs independently of the pooled screening approach (Fig. 3C). Although we did not try to validate them, the Pumilio homologues (*PUM1* and *PUM2*) were consistently enriched for roles in mediating resistance to paraquat in both conditions at day 21 (Fig. 3A, B). Our genetic screens demonstrate that RBPs convey both resistance and sensitivity to paraquat. We decided to focus on RBP knockouts that conveyed a competitive advantage during oxidative stress.

### Translation initiation factors as regulators of sensitivity to paraquat toxicity

**FIG. 4.**
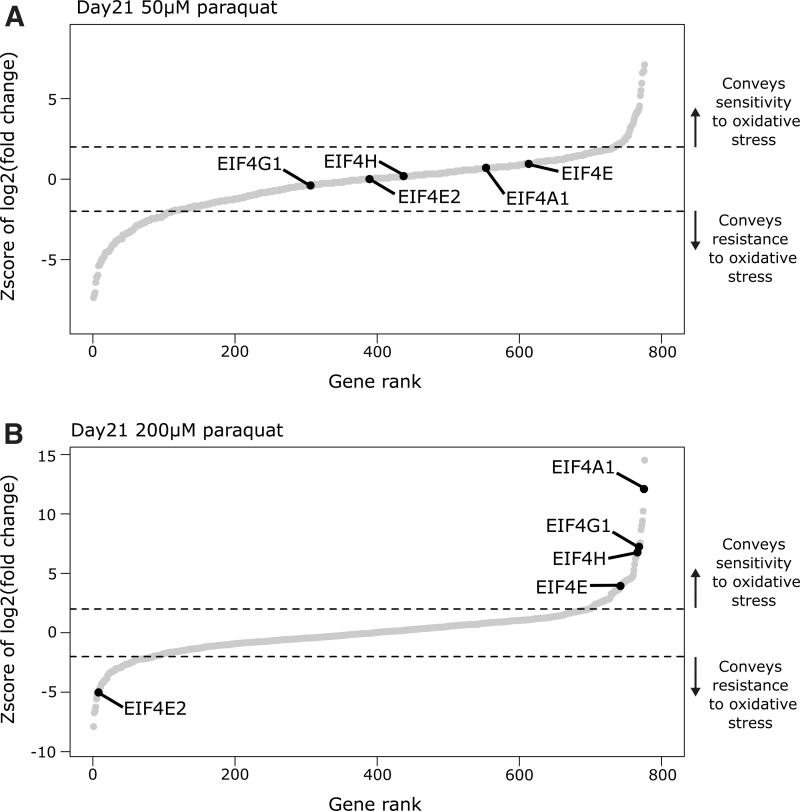
Translation initiation factors as regulators of sensitivity to paraquat toxicity. **(A)** Enrichment of RBPs in 50 μM paraquat screen compared to the condition without paraqaut treatment at day 21. Select RBPs highlighted. **(B)** Enrichment of RBPs in 200 μM paraquat screen compared with the condition without paraqaut treatment at day 21. Select RBPs highlighted. Dashed line represents 2 standard deviations of the distribution of nontargeting sgRNAs from the mean representation of nontargeting sgRNAs. This is the cutoff for statistical significance.

In addition to a role in supporting Jurkat proliferation or survival in the absence of paraquat (Supplementary Table S8**)**, translation initiation factors enriched for roles in conveying sensitivity and resistance to oxidative stress. eIF4A1, a key RNA helicase of the eIF4F translation initiation complex, was the second ranked hit conveying sensitivity at high 200 μM paraquat concentration, whereas it did not enrich in the low 50 μM paraquat concentration screen (Fig. 4). The 10 *eIF4A1* sgRNAs had concordant enrichment in our screen (Supplementary Fig. S3A), and this result was validated with three independent gRNAs (Supplementary Fig. S3B**)**.

Moreover, eIF4G1 a component of the eIF4F complex and eIF4H an accessory factor for eIF4A1 had the same pattern of enrichment (Fig. 4). This is consistent with the expectation that the paraquat concentrations used represent low- and high-oxidative stress-inducing conditions and that global inhibition of translation initiation is increasingly critical for cell survival as oxidative stress increases. This is further supported by the enrichments of the eIF4E1 and eIF4E2 paralogues. eIF4E1 is enriched for a role in mediating sensitivity to oxidative stress solely in the high paraquat screen (Fig. 4B). By contrast, eIF4E2 is enriched for a role in conveying resistance to the high paraquat concentration (Fig. 4B). These enrichments were validated with three independent guides for both *eIF4E1* and *eIF4E2* (Supplementary Fig. S3B**)**. These observations are consistent with a previous study suggesting that eIF4E1 promotes translation initiation, whereas eIF4E2 has a role in blocking translation initiation.^21^

In high paraquat concentrations, eIF4A1 had stronger enrichment for a role conveying sensitivity to oxidative stress than eIF4H or eIF4G1, which showed similar enrichments to each other (Fig. 4B). This may be due to an additional role for eIF4A1, besides mediating translation initiation, in limiting the formation of SGs by reducing RNA condensation.^13^

### RBPs promote sensitivity to low and resistance to high paraquat concentrations

**FIG. 5.**
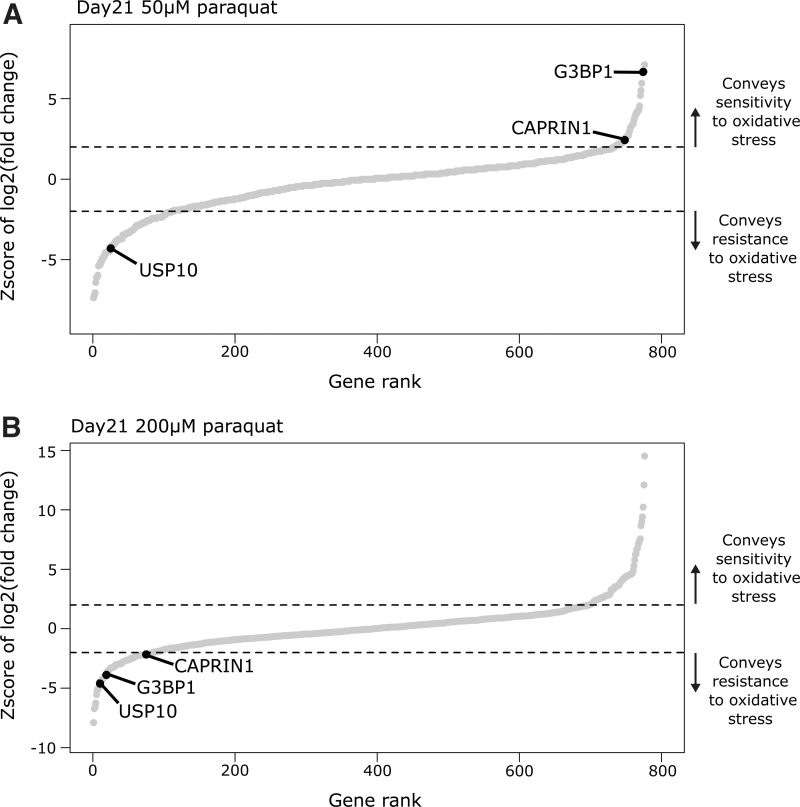
RBP promoting sensitivity to low and resistance to high paraquat concentrations. **(A)** Enrichment of RBPs in 50 μM paraquat screen compared with the condition without paraqaut treatment at day 21. Select RBPs highlighted. **(B)** Enrichment of RBPs in 200 μM paraquat screen compared with the condition without paraqaut treatment at day 21. Select RBPs highlighted. Dashed line represents 2 standard deviations of the distribution of nontargeting sgRNAs from the mean representation of nontargeting sgRNAs. This is the cutoff for statistical significance.

At the low 50 μM paraquat concentration, *G3BP1* knockout was the third hit mediating sensitivity to oxidative stress (Fig. 5A). However, at the high 200 μM paraquat concentration, *G3BP1* knockout was greatly enriched in the opposite direction, indicating it mediated resistance (Fig. 5B). All sgRNAs targeting *G3BP1* had the same pattern of enrichment (Supplementary Fig. S4A), and this result was validated with three individual gRNAs independently of the pooled screening approach (Supplementary Fig. S4B). Moreover, this trend was also true for CAPRIN1, a known interacting partner of G3BP1 (Fig. 5).^16^ USP10, another G3BP1 binding partner, strongly enriched as mediating resistance to oxidative stress in our genetic screens (Fig. 5). In addition to an RNA binding capacity, USP10 acts as a deubiquitinase, and has previously been inferred to have antioxidant function.^15^ In the low-oxidative stress condition, G3BP1 may sensitize cells to oxidative stress by limiting USP10 antioxidant function; however, in the high-oxidative stress condition, G3BP1 along with CAPRIN1 may provide resistance by supporting SG formation.

## Discussion

The results presented here validate the utility of a targeted sgRNA library for the study of human RBPs by CRISPR-Cas9-mediated gene knockout. The robustness of sgRNA library as a reagent for performing genetic screens is evident from the normal and tight distribution of sgRNAs in the library and from the enrichment of control genes according to our expectations in the paraquat toxicity screens. In comparison with genome-wide sgRNA libraries, our targeted RBP sgRNA library is an order of magnitude smaller. This enables it to be readily employed in genetic screens, particularly those where maintaining enough cells to adequately represent a genome-wide sgRNA library is difficult or impossible. These include screens performed with primary cells or screens that require the physical separation of cells such as by flow cytometry. Furthermore, cells transduced with our sgRNA library can be selected by various methods including antibiotic selection, and either fluorescent or magnetic assisted cell sorting. Moreover, the cell surface transduction marker can be bound by antibodies conjugated to a range of fluorophores enabling its integration in established assays.

A previous genetic screen of paraquat-induced oxidative stress in Jurkat cells using a library targeting ∼3000 genes related to metabolism identified cytochrome P450 oxidoreductase as initiating the production of ROS through the redox cycling of paraquat.^22^ The library targeted 50 metabolic enzymes that have “moonlighting” functions as RBPs none of which were significantly enriched. Thus, our approach offered the potential to identify novel regulators of paraquat toxicity and among these were the Pumilio proteins that conveyed resistance to oxidative stress. These RBPs facilitate the translational repression, deadenylation, and subsequent decay of target transcripts through recruitment of the CCR4-NOT complex.^23,24^ A yeast Pumilio orthologue has previously been described as mediating the translational repression of genes linked to oxidative stress.^25^ Furthermore, it is known that upon oxidative stress, mammalian Pumilio proteins, which are structurally conserved but functionally divergent with their yeast orthologues, localize to SGs.^23^ However, a functional role for mammalian Pumilio proteins in redox biology has not been described. It is possible they play a role in mitochondrial function or DNA damage responses after oxidative stress.^26,27^

The screen identified a role for the binding partners CSDE1 and STRAP in conveying sensitivity to oxidative stress. They have been shown to bind to each other and to promote erythropoiesis.^28–30^ Their mechanism of action is unclear, but they may affect the expression of many genes including those that have roles in promoting the translation of transcripts encoding ribosomal proteins and translation initiation factors as well as factors involved in the mitochondrial respiratory chain.^29–31^ These RBPs may have roles in redox biology and provide an additional layer of regulation over certain signaling pathways in lymphocytes; for example, PI3(K) signaling upon B cell or T cell activation. Alternatively, they may have broader roles regulating cellular stress tolerance, for example, endoplasmic reticulum stress experienced by plasma cells during the rapid production of antibodies.

STRAP has previously been identified as conveying sensitivity to hydrogen peroxide-mediated oxidative stress by a genome-wide genetic screen in K562 cells.^32^ However, Pumilio proteins and CSDE1 were not found in that study. Furthermore, in a genome-wide genetic screen of cell fitness in high or low oxygen conditions, CSDE1 and STRAP enriched for roles in promoting K562 cell proliferation or survival in hypoxic conditions.^33^ Together, these observations prompt the hypothesis that CSDE1 and STRAP support the production of ROS by cellular metabolism. Such a role might support cell viability in hypoxia, sensitize cells to oxidative stress, and potentiate cell signaling pathways.

Factors involved in promoting translation initiation enriched for roles in conveying sensitivity to paraquat but only at the higher concentration used here. This demonstrates that a robust inhibition of translation initiation makes Jurkat cells more competitive during chronic exposure to high amounts of oxidative stress. In turn, this implies that unmodified Jurkat cells retain some degree of global translation during chronic stress to their detriment; perhaps such a response enables their competitive recovery to transient stress exposure. In our genetic screen at the higher concentration of paraquat, eIF4A1 and G3BP1 were greatly enriched in opposing directions. We speculate that this indicates SGs may themselves convey resistance to oxidative stress rather than solely being a consequence of global translational inhibition.

## Materials and Methods

### Construction of sgRNA vector backbone

PKLV2_hU6_BbsI-ccdB-BbsI_iScaffold_mPGK_puro-2A-CD90.1 was generated in two rounds of cloning. First, a polymerase chain reaction (PCR) product encoding CD90.1 and the appropriate flanking sequences were generated and ligated (Gibson assembly) into a \*Xho*I + \*Not*I linearized vector (Addgene plasmid 67974). Second, a PCR product (template: pcDNA-DEST47) encoding the ccdB toxic gene and the appropriate flanking sequences were generated and ligated (Gibson assembly) into the \*Bbs*I linearized intermediate vector. For individual sgRNAs, two 24 nt oligonucleotides were annealed and ligated (T4) into the \*Bbs*I linearized backbone vector.

### Generation of targeted human RBP CRISPR-Cas9 sgRNA library

The Broad Institute online web portal “sgRNA designer” was used to generate sgRNA designs against target genes.^34^ The first nucleotide of each 20 nt seed region was invariantly replaced with guanine. An oligonucleotide pool (Twist Bioscience) was converted to dsDNA by PCR and ligated (Gibson assembly) into our backbone vector at a molar ratio of 7.5:1. The product was transformed in electrocompetent bacteria (ElectroMAX™ Stbl4™ #11635018; Thermo Scientific™) following the manufacturer's protocol. Transformed bacteria were plated on 24.5 cm^2^ LB-AMP plates (CLS431272-16EA; Corning^®^) and incubated at 32°C for 20 h. The bacteria were harvested with help of LB broth medium and a razor blade. Then, the plasmid DNA was isolated (12162; Qiagen^®^), the manufacture's protocol was followed.

### Cell culture

HEK293FT cells were maintained in supplemented Dulbecco's modified Eagle's high glucose medium (41965; Gibco™) with 10% fetal bovine serum (FBS). Jurkat cells were maintained in supplemented Roswell Park Memorial Institute 1640 (RPMI-1640) medium with 10% FBS and 1 × GlutaMAX™ (35050061; Gibco).

### Lentiviral transduction

HEK293FT cells in logarithmic growth phase were seeded in dishes (Nunc™ 150350). The next day, a transfection mixture of 1000 μL OptiMEM (31985; Gibco™), 30 μL TransIT^®^-293 (MIR2700; Mirus^®^ Bio), 9 μg transfer vector, 4.5 μg packaging vector (pΔR8.2), and 4.5 μg envelope vector (pMΔG) was added dropwise and incubated with the cells overnight. The medium was replaced. Then, for two sequential days, the viral supernatant was harvested. Jurkat cells were transduced with VSV-G pseudotyped lentivirus in the presence of 4 ng/mL polybrene (H9268; Sigma-Aldrich^®^), by spinfection at 1500 *g* for 99 min at 32°C.

### CRISPR-Cas9 genetic screen of oxidative stress

Clonal Jurkat-Cas9 cells were transduced with the human RBP sgRNA library. Transduced cells were selected with 0.75 μg/mL puromycin from day 2 to day 5 post-transduction. On day 6, cells were cultured in 0, 50, or 200 μM paraquat (856177; Sigma-Aldrich^®^) for 14 or 21 days. Cells were harvested at the start and end of paraquat exposure. The representation of the library was maintained at >1000 cells per sgRNA in all conditions.

### Next-generation sequencing library generation

Genomic DNA (gDNA) was isolated as previously described.^35^ Next-generation sequencing (NGS) libraries were generated in one round by PCR from 2.5 μg gDNA in 22 cycles with Q5 polymerase following the manufacturer's protocol. PCR products were concentrated (D4031; Zymo Research), size was selected by gel electrophoresis, and purified (D4005; Zymo Research). Multiplexed NGS libraries were sequenced with an Illumina™ HiSeq with a 75 bp paired end read. DESeq was used to determine the abundance of sgRNAs from raw fastq files.^36^ Analysis of our genetic screens was performed with the MAGeCK software.^37^

## Supplementary Material

Supplemental data
