# Supplementary figures and images for: RNA Binding Proteins As Regulators of Oxidative Stress Identified by a Targeted CRISPR-Cas9 Single Guide RNA Library
Source: CRISPR J. 2021 Jun 16;4(3):427–37. doi: 10.1089/crispr.2020.0116 (PMC8236562; doi:10.1089/crispr.2020.0116)

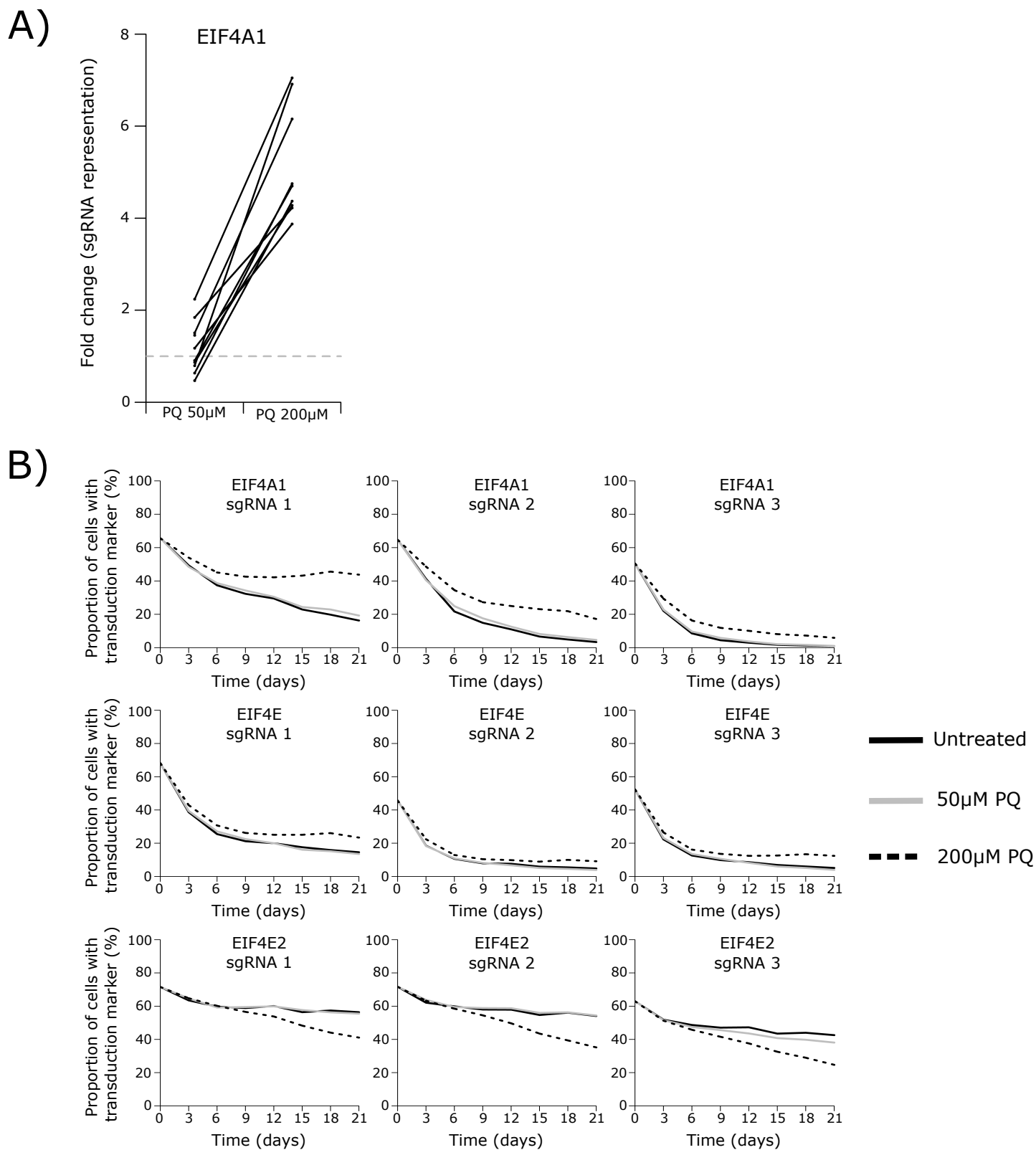

Supplement: Supplemental data [file Supp_Tables-Figures.zip › Supp_FigS3.pdf]

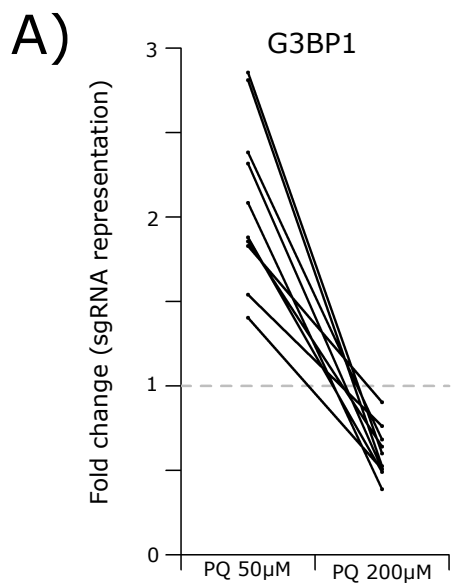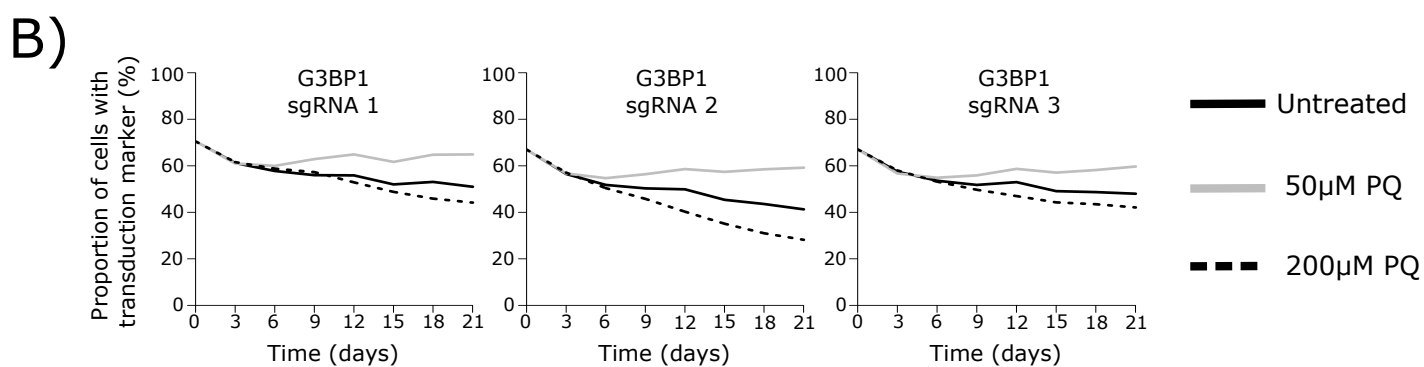

Supplement: Supplemental data [file Supp_Tables-Figures.zip › Supp_FigS4.pdf]

## Jurkat Cas9

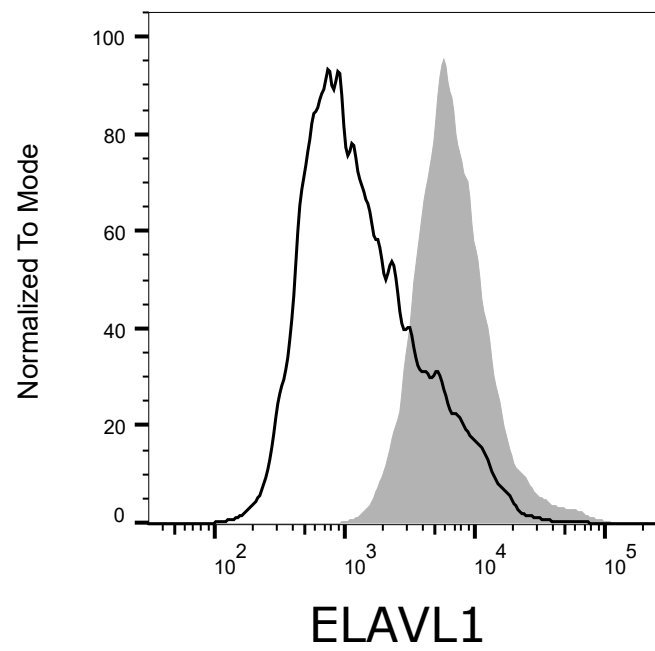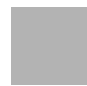

Non-targetting sgRNA

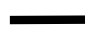

ELAVL1 sgRNA

Supplement: Supplemental data [file Supp_Tables-Figures.zip › Supp_FigS1.pdf]

A)

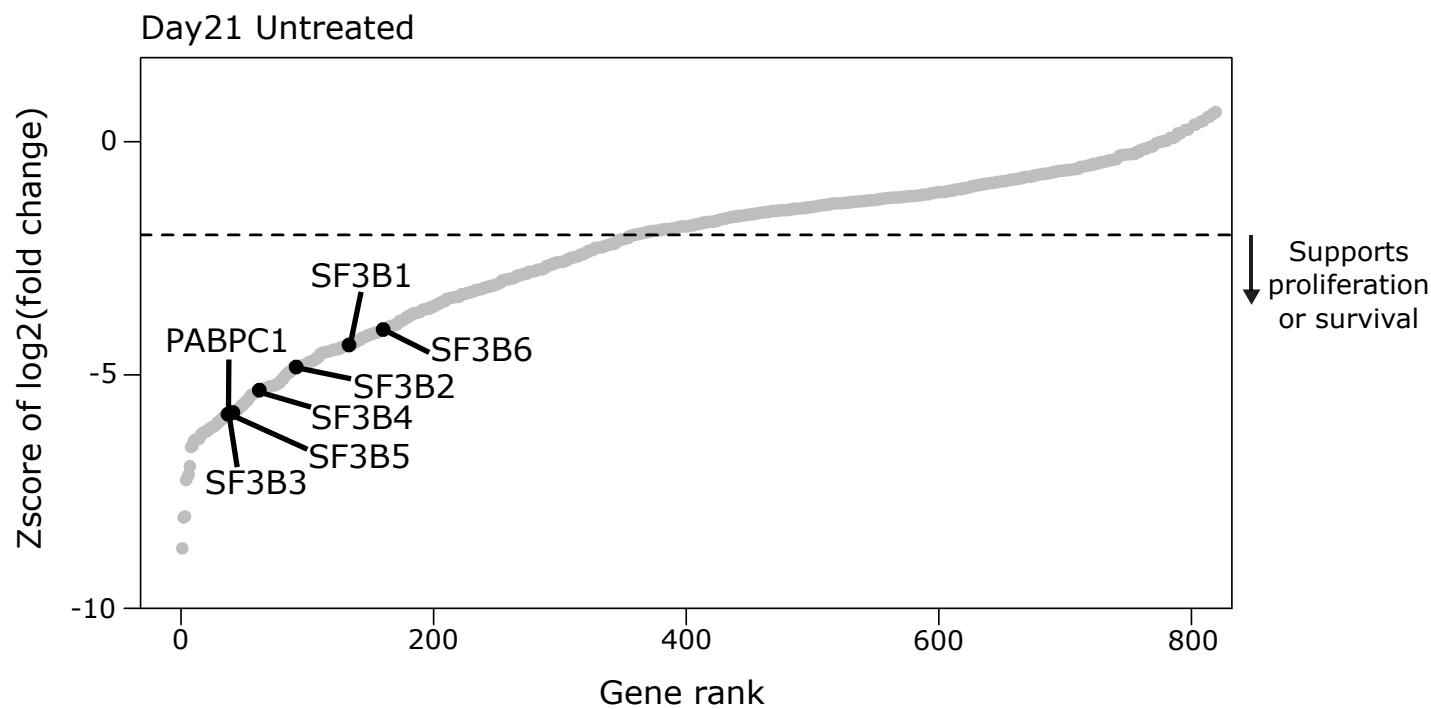

B)

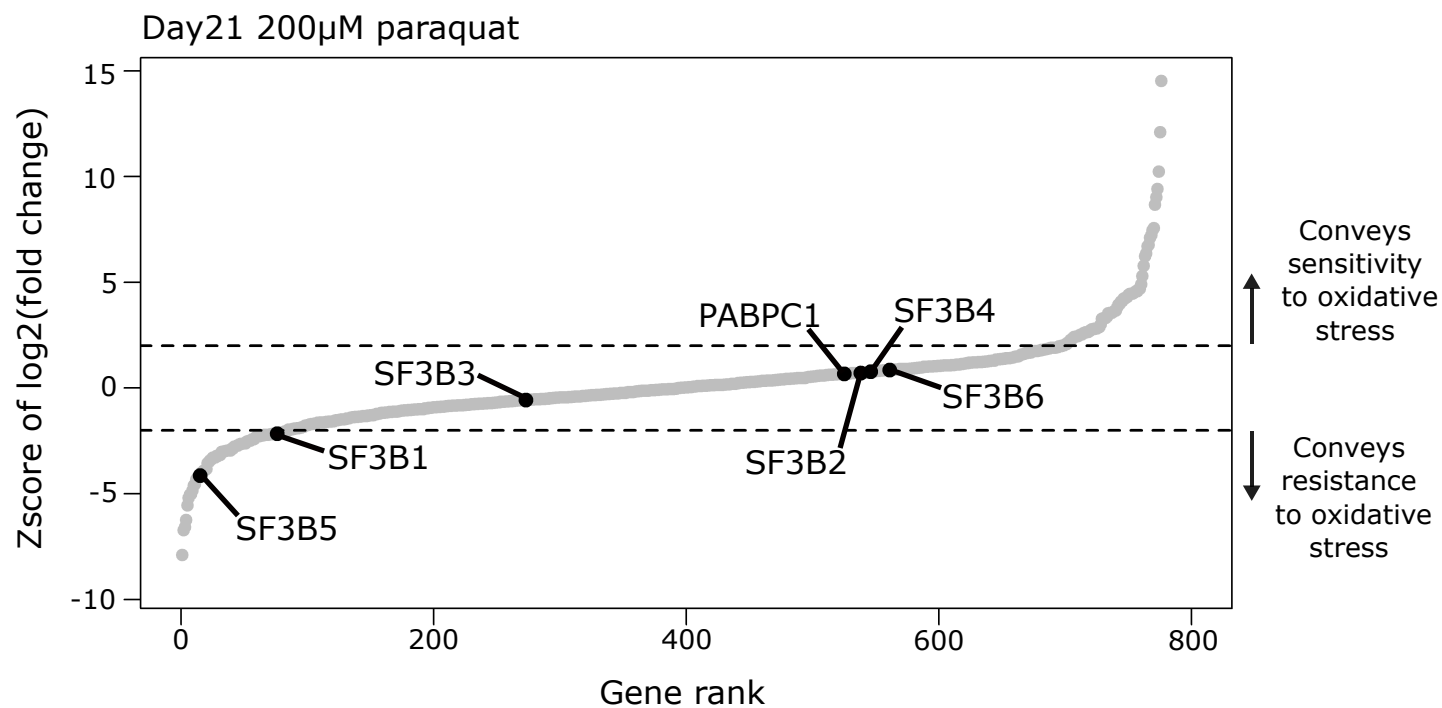

Supplement: Supplemental data [file Supp_Tables-Figures.zip › Supp_FigS2.pdf]
