# Supplementary material for: RNA Binding Proteins As Regulators of Oxidative Stress Identified by a Targeted CRISPR-Cas9 Single Guide RNA Library
Source: CRISPR J. 2021 Jun 16;4(3):427–37. doi: 10.1089/crispr.2020.0116 (PMC8236562; doi:10.1089/crispr.2020.0116)
Supplement: Supplementary file 5 [file Supp_Tables-Figures.zip › Supplementary legends.odt]

Supplementary Figure 1. Validation of Jurkat-Cas9 genome editing.

Flow cytometry analysis of Jurkat-Cas9 cells transduced with a sgRNA targeting ELAVL1 or a non-targeting (NT) control.

Supplementary Figure 2. RBPs that regulate proliferation or survival of Jurkat cells are not a source of false positives.

A) Enrichment of RBPs in 0µM paraquat at day21 compared to the day0 condition. Select RBPs highlighted.

B) Enrichment of RBPs in 200µM paraquat screen compared to the condition without paraqaut treatment at day21. Select RBPs highlighted.

Dashed line represents two standard deviations of the distribution of non-targeting sgRNAs from the mean representation of non-targeting sgRNAs. This is the cutoff for statistical significance. 

Supplementary Figure 3.  Validation of eIF4A1, eIF4E1 and eIF4E2 as regulators of oxidative stress

A) Fold change of eIF4A1 sgRNAs between either the day 21 50µM and 200µM datasets and the day 21 0µM condition. Dashed line represents no change. Connecting lines indicate the enrichment of the individual sgRNAs in both conditions.

B) Validation that the genetic knockout of eIF4A1 or eIF4E1 in Jurkat cells conveys resistance to paraquat at 200µM. Validation that the genetic knockout of eIF4E2 in Jurkat cells conveys resistance to paraquat at 200µM.

Supplementary Figure 4. Validation of G3BP1 as a regulator of oxidative stress

A) Fold change of G3BP1 sgRNAs between either the day 21 50µM and 200µM datasets and the day 21 0µM condition. Dashed line represents no change. Connecting lines indicate the enrichment of the individual sgRNAs in both conditions.

B) Validation that the genetic knockout of G3BP1 in Jurkat cells conveys resistance to paraquat at 50µM and sensitivity to paraquat at 200µM.
